# Cushing’s Disease: Assessment of Early Cardiovascular Hemodynamic Dysfunction With Impedance Cardiography

**DOI:** 10.3389/fendo.2021.751743

**Published:** 2021-10-01

**Authors:** Agnieszka Jurek, Paweł Krzesiński, Grzegorz Gielerak, Przemysław Witek, Grzegorz Zieliński, Anna Kazimierczak, Robert Wierzbowski, Małgorzata Banak, Beata Uziębło-Życzkowska

**Affiliations:** ^1^ Department of Cardiology and Internal Diseases, Military Institute of Medicine, Warsaw, Poland; ^2^ Department of Internal Medicine, Endocrinology and Diabetes, Medical University of Warsaw, Warsaw, Poland; ^3^ Department of Neurosurgery, Military Institute of Medicine, Warsaw, Poland

**Keywords:** Cushing’s disease, impedance cardiography, cardiovascular complications, arterial hypertension, left ventricular systolic dysfunction

## Abstract

**Background:**

Cushing’s disease is a rare condition associated with a high cardiovascular risk and hypercortisolemia-related hemodynamic dysfunction, the extent of which can be assessed with a noninvasive method, called impedance cardiography. The standard methods for hemodynamic assessment, such as echocardiography or ambulatory blood pressure monitoring may be insufficient to fully evaluate patients with Cushing’s disease; therefore, impedance cardiography is being currently considered a new modality for assessing early hemodynamic dysfunction in this patient population. The use of impedance cardiography for diagnosis and treatment of Cushing’s disease may serve as personalized noninvasive hemodynamic status assessment and provide a better insight into the pathophysiology of Cushing’s disease. The purpose of this study was to assess the hemodynamic profile of Cushing’s disease patients and compare it with that in the control group.

**Material and Methods:**

This observational prospective clinical study aimed to compare 54 patients with Cushing’s disease (mean age 41 years; with 64.8% of this population affected with arterial hypertension) and a matched 54-person control group (mean age 45 years; with 74.1% of this population affected with arterial hypertension). The hemodynamic parameters assessed with impedance cardiography included the stroke index (SI), cardiac index (CI), systemic vascular resistance index (SVRI), velocity index (VI), (ACI), Heather index (HI), and thoracic fluid content (TFC).

**Results:**

The Cushing’s disease group was characterized by a higher diastolic blood pressure and a younger age than the control group (82.9 *vs.* 79.1 mmHg, p=0.045; and 41.1 *vs.* 44.9 years, p=0.035, respectively). Impedance cardiography parameters in the Cushing’s disease group showed: lower values of SI (42.1 *vs.* 52.8 ml/m^2^; p ≤ 0.0001), CI (2.99 *vs.* 3.64 l/min/m^2^; p ≤ 0,0001), VI (42.9 *vs.* 52.1 1/1000/s; p=0.001), ACI (68.7 *vs.* 80.5 1/100/s^2^; p=0,037), HI (13.1 *vs.* 15.2 Ohm/s^2^; p=0.033), and TFC (25.5 *vs.* 27.7 1/kOhm; p=0.006) and a higher SVRI (2,515 *vs.* 1,893 dyn*s*cm^-5^*m^2^; p ≤ 0.0001) than those in the control group.

**Conclusions:**

Cushing’s disease is associated with significantly greater vasoconstriction and left ventricular systolic dysfunction. An individual assessment with impedance cardiography may be useful in Cushing’s disease patients in order to identify subclinical cardiovascular complications of chronic hypercortisolemia as potential therapeutic targets.

## Introduction

Cushing’s disease is a rare chronic disorder due to excessive secretion of adrenocorticotropic hormone (ACTH) by a pituitary adenoma. Cushing’s disease-associated hypercortisolemia has been linked to significant functional and structural systemic abnormalities, with changes in the hemodynamic profile and a considerably increased cardiovascular risk (1–3). Some adverse effects of chronic hypercortisolemia include hemodynamic disturbances associated with excessive vascular constriction and elevated blood pressure (BP), obesity, impaired carbohydrate metabolism, and dyslipidemia, all of which may contribute to substantial cardiovascular remodeling (4–7). Subclinical effects of hypercortisolemia may be undetectable with standard hemodynamic assessment methods. Therefore, novel diagnostic tools should be sought to help detect abnormalities in Cushing’s disease patients as early as possible and improve the chances of their optimal targeted treatment and, as a result, lower the cardiovascular risk. One noninvasive and well-validated tool for assessing cardiovascular hemodynamics is impedance cardiography, which helps assess such cardiovascular hemodynamic parameters as arterial stiffness, intravascular volume, and cardiac function, which are useful in clinical evaluation of Cushing’s disease patients, particularly those with concomitant arterial hypertension (8–11). Therefore, the purpose of this study was to use this method for assessing cardiovascular function to evaluate the hemodynamic profiles of patients with Cushing’s disease and compare them with those in the control group.

## Materials and Methods

### Study Population

Two age-matched groups were compared in this observational, prospective clinical study. The Cushing’s disease group comprised 54 patients with Cushing’s disease (including 12 males; mean age 41 years; with 64.8% of this population with controlled arterial hypertension – mean blood pressure 126/83 mmHg), and the control group comprised 54 individuals (including 19 males; mean age 45 years; with 74.1% of this population with controlled arterial hypertension − mean blood pressure 121/79 mmHg). Neither study group included patients with significant comorbidities.

The study was conducted in accordance with the Declaration of Helsinki and Good Clinical Practice (GCP). The study protocol had been approved by the Bioethics Committee of Military Institute of Medicine in Warsaw. All study participants had provided their written informed consent.

### Cushing’s Disease

The Cushing’s disease group included both male and female patients newly diagnosed with Cushing’s disease, defined based on a standard hormone blood test and imaging study results in accordance with the European Society of Endocrinology guidelines: symptoms of hypercortisolemia combined with serum cortisol suppression or a decrease in urinary free cortisol by >50% during a high-dose dexamethasone suppression test (HDDST; 8 mg over 48 h) or a positive ACTH stimulation test with the use of corticotropin-releasing hormone (CRH; 100 mg intravenously) and evidence of pituitary adenoma in magnetic resonance imaging (MRI) (12). Inferior petrosal sinus sampling was performed in all cases of a microadenoma smaller than 6 mm, inconclusive MRI results, or contradictory responses to dynamic testing. All patients from the Cushing’s disease group underwent standard hormone level tests for ACTH, follicle-stimulating hormone, luteinizing hormone, and thyroid-stimulating hormone and history-taking for any concomitant impaired carbohydrate metabolism (type 2 diabetes mellitus, impaired fasting glycemia, and impaired glucose tolerance) diagnosed previously or during the first study visit. Since none of the Cushing’s disease group patients had been taking any drugs affecting the hypothalamic–pituitary–adrenal axis, their medical treatment had no effect on their hemodynamic assessments.

### Control Group

The control group-from which a subgroup of 54 individuals, matched for key clinical variables (age, sex, body mass index (BMI), mean blood pressure (MBP), proportion of arterial hypertension cases), had been selected for a comparative analysis—comprised the subjects from the government-funded study “Non−invasive hemodynamic assessment in hypertension (FINE-PATH)” (ClinicalTrials.gov Identifier NCT01996085)*, conducted at the Military Institute of Medicine.* There were 120 initially recruited patients of both sexes, with arterial hypertension treated for at least 12 months, and 35 healthy individuals of both sexes, without cardiovascular conditions or any other clinically significant internal medical conditions.

### Exclusion Criteria

The study exclusion criteria were conditions significantly affecting cardiovascular system function and those that could confound the obtained results, namely: coronary artery disease, including history of myocardial infarction; chronic heart failure with mid-range ejection fraction and heart failure with reduced ejection fraction, with left ventricular ejection fraction <50%; history of pulmonary embolism; documented history of stroke or transient ischemic attack; severe chronic obstructive pulmonary disease, with the Tiffeneau index (or forced expiratory volume in 1 second expressed as percentage of vital capacity, FEV1) *<*50% of the predicted value; respiratory failure (partial pressure of oxygen [PaO2] in blood <60 mmHg and/or increased partial pressure of carbon dioxide [PaCO2] >45 mmHg); status post head injury; pregnancy; lack of consent; any conditions making the patient unable to follow the study protocol.

### Clinical Study

The clinical examination was conducted with a particular focus on cardiovascular risk factors (including family history of cardiovascular disease; cardiovascular symptoms; comorbidities; nicotine dependence; impaired carbohydrate metabolism; lifestyle; office BP measurement, including systolic and diastolic blood pressure (SBP and DBP); heart rate (HR); and anthropometric measurements (height, body weight, BMI). The office BP measurement was performed with the use of an automatic device (Omron M4 Plus, Japan) in accordance with European Society of Cardiology guidelines (13).

### Impedance Cardiography

Hemodynamic parameters were measured at rest in a supine position *via* impedance cardiography with a *Niccomo*™ device (Medis, Ilmenau, Germany) during a 10-minute assessment. These 10-minute impedance cardiography recordings were used to analyze (*Niccomo Software*) in detail the mean hemodynamic parameters, such as HR [bpm]; SBP [mmHg], DBP [mmHg]; stroke volume (SV) [ml]; stroke index (SI) [ml/m^2^], cardiac output (CO) [l/min]; cardiac index (CI) [l/min/m^2^]); systemic vascular resistance (SVR) [dyn*s*cm^−5^]; systemic vascular resistance index (SVRI) [dyn*s*cm^−5^*m²]; velocity index (VI) [1/1000/s]: VI=1000*dZmax*Z0^−1^, expressing peak aortic flow velocity; acceleration index (ACI) [1/100/s^2^]: ACI=100*dZmax*dt^−1^, expressing peak aortic flow acceleration; Heather index (HI) [Ohm/s^2^]: HI=dZmax*TRC, expressing the ratio of peak systolic outflow to the time interval from the Q/R wave peak in ECG to the impedance cardiography wave peak and reflecting both the cardiac inotropic function and thoracic fluid content (TFC) [1/kOhm]. According to the data obtained from the PREDICT study, which helped identify the different risk groups based on the stroke index and thoracic fluid content values, for our study we adopted the cutoff values of SI at <35 ml/m^2^ and of TFC at >35 1/kOhm (14).

Impedance cardiography is a non-invasive method designed for monitoring hemodynamic parameters on the basis of analysis of thoracic electrical resistance. During the examination, voltage changes associated with changes in blood volume and velocity in large vessels during systole and diastole are analyzed. This enables the calculation of parameters, including stroke volume and cardiac output, which is a particular advantage of the method. This method has also some limitations. The diagnostic value of the impedance cardiography is questionable in the following clinical situations: tachycardia> 250/min, significant arrhythmias, severe aortic regurgitation, extremely high blood pressure, intra-aortic counterpulsation, severe septic shock, post-sternotomy condition, very short or tall stature, severe obesity or severe malnutrition. Moreover, the quality of measurement strongly depends on skin preparation and movement artifacts (8).

### Statistical Methods

Electronic filing and statistical analysis of data were conducted with *MS Office Excel 2016* and *Statistica 12.0* software (*StatSoft Inc., Tulsa, USA*). Continuous variables were expressed as means ± standard deviation (SD), medians, and interquartile ranges, and categorical (qualitative) variables were expressed as counts (n) and proportions (%). Continuous variable distribution was assessed visually with the Shapiro–Wilk test. For each comparative analysis, the propensity score matching method was used to select from the control group a subgroup matched for key clinical variables (age, sex, BMI, MBP, arterial hypertension rates), which may have a considerable impact on the evaluated variables. The differences in absolute values of normally distributed continuous variables were analyzed with a t-test, and the Mann–Whiney U test was used for the variables that were not normally distributed. Categorical (qualitative) variables were analyzed with the chi-square and Fisher’s tests. Statistical significance was adopted at p <0.05.

## Results

### Demographic and Clinical Data of the Cushing’s Disease Group

Detailed patient characteristics of the Cushing’s disease group are presented in **Table 1**. Arterial hypertension rates were similar in the Cushing’s disease and control groups (64.8% *vs.*74.1%; p=0.296), with all arterial hypertension patients undergoing medical treatment, usually with one or two antihypertensive drugs. Over 70% of Cushing’s disease patients had abnormal body weight, with 22 patients obese (40.7%). A total of 20 out of 54 patients with Cushing’s disease (37%) had been diagnosed with type 2 diabetes mellitus, 5 (9.3%) with prediabetes, and 29 (46.3%) exhibited normal glucose tolerance. Out of Cushing’s disease patients with type 2 diabetes mellitus, 14 had been receiving metformin, 5 metformin and insulin, and 1 insulin. Forty-three out of the 54 patients with Cushing’s disease had normal anterior pituitary lobe function. Eleven patients with an invasive corticotropic-releasing tumor had thyroid-stimulating hormone deficiency, but it was well controlled with a stable dose of L-thyroxin.

**Table 1:** Comparison of the control group and Cushing’s disease group in terms of patient characteristics and the hemodynamic parameters assessed with impedance cardiography.

VARIABLES	Controls mean ± SD	Patients with Cushing’s disease means ± SD	p-value
**PATIENT CHARACTERISTICS**			
**Age [years]**	44.9 ± 9.4	41.1 ± 13.5	**0.035**
**Male sex, n [%]**	19/54 (35.2)	12/54 (22.2)	0.136
**Body mass index [kg/m^2^]**	29.2 ± 4.9	29.7 ± 6.5	0.988
**Heart rate [bpm]**	69.8 ± 10.1	71.9 ± 11.4	0.322
**Systolic blood pressure [mmHg]**	121.6 ± 10.1	126.1 ± 16.8	0.096
**Diastolic blood pressure [mmHg]**	79.1 ± 8.0	82.9 ± 11.6	**0.045**
**Arterial hypertension, n [%]**	40/54 (74.1)	35/54 (64.8)	0.296
**Creatinine [mg/dL]**	0.82 ± 0.15	0.87 ± 0.27	0.836
**Left ventricular ejection fraction [%]**	66,69 ± 3,27	65,8 ± 4,2	0.323
**IMPEDANCE CARDIOGRAPHY**			
**Heart rate [bpm]**	70.2 ± 11.8	71.4 ± 11.9	0.581
**Systolic blood pressure [mmHg]**	121.2 ± 11.1	125.6 ± 17.0	0.113
**Diastolic blood pressure [mmHg]**	78.9 ± 9.0	82.5 ± 11.7	0.076
**Mean blood pressure [mmHg]**	89.9 ± 9.1	93.3 ± 12.0	0.093
**Pulse pressure [mmHg]**	42.3 ± 6.5	43.2 ± 11.2	0.507
**Stroke index [ml/m^2^]**	52.8 ± 9.0	42.1 ± 10.4	**<0.0001**
**Cardiac index [l/min/m^2^]**	3.64 ± 0.61	2.99 ± 0.78	**<0.0001**
Systemic vascular resistance index [dyn*s*cm^−5^*m²]	1,893 ± 379.1	2,515 ± 737.7	**<0.0001**
**Velocity index [1/1000/s]**	52.1 ± 14.9	42.9 ± 13.4	**0.001**
**Acceleration index [1/100/s^2^]**	80.5 ± 31.6	68.7 ± 25.7	**0.037**
**Heather index [Ohm/s^2^]**	15.2 ± 4.7	13.1 ± 5.0	**0.033**
**Thoracic fluid content [1/kOhm]**	27.7 ± 4.1	25.5 ± 3.9	**0.006**
**Stroke index < 35 ml/m^2^, n [%]**	2/54 (3.7)	14/51 (27.5)	**0.001**
**Thoracic fluid content >35 1/kOhm, n [%]**	2/54 (3.7)	0/52 (0)	0.161

SD, standard deviation.

In bold: Statistical significance was adopted at p < 0.05.

### Hemodynamic Data of the Cushing’s Disease Group

Impedance cardiography parameters in the Cushing’s disease group and the common abnormalities in impedance cardiography assessments are presented in **Table 1**. During impedance cardiography, the Cushing’s disease group had an average blood pressure of 126/83 mmHg, mean blood pressure of 93 mmHg, and a mean heart rate of 71 bpm. Fourteen patients (27.5%) had a low stroke index (<35 ml/m^2^). No patients from the study group showed increased thoracic fluid content (of >35 1/kOhm).

### A Comparison Between Cushing’s Disease Patients and Controls in Terms of Patient Characteristics and Hemodynamic Parameters Assesses *via* Impedance Cardiography

**Table 1** compares patient characteristics in the Cushing’s disease and control groups. The average blood pressure was normal in both groups. The study groups differed significantly only in terms of patient age and diastolic blood pressure values. Despite only slight differences in the evaluated key hemodynamic parameters (heart rate, systolic blood pressure, diastolic blood pressure), a comparison of the remaining impedance cardiography variable values demonstrated a number of differences between the study groups. Impedance cardiography showed markedly lower values of cardiac function in patients with Cushing’s disease in comparison with those in controls, namely a lower stroke index (p < 0.0001) and cardiac index (p < 0.0001); significantly lower indices of myocardial contractility, namely velocity index (p = 0.001), acceleration index (p = 0.037), Heather index (p = 0.033); a significantly lower thoracic fluid content (p = 0.006); and a significantly higher systemic vascular resistance index (p < 0.0001). A total of 3.7% of controls and 27.5% of patients with Cushing’s disease had a low stroke index (< 35 ml/m^2^; p = 0.001). There were no statistically significant differences between the study groups in terms of the other evaluated parameters.

## Discussion

This study demonstrated hemodynamic abnormalities in patients with newly diagnosed Cushing’s disease, despite an optimal blood pressure control in most of them. Comprehensive hemodynamic assessments with impedance cardiography showed that the hemodynamic profile of Cushing’s disease patients differs from that of individuals without hypercortisolemia. We would like to emphasize that the Cushing’s disease patients included in this study had no clinically overt cardiovascular dysfunction, and patients with severe comorbidities were excluded.

The demographic, history-related, and cardiovascular function data in our study differed from those obtained in other studies on cardiovascular dysfunction in Cushing’s disease patients (15–17). This was due to the fact that the patients recruited to those other studies had not been selected; instead, they were patients with Cushing’s disease of various duration and at various stages of treatment. We would like to emphasize that our thorough cardiovascular hemodynamics assessment with impedance cardiography is one of the first attempts of utilizing this modality in patients with Cushing’s disease.

Our hemodynamic assessment with impedance cardiography showed significantly decreased cardiac function, indicators of myocardial contractility, and thoracic fluid content, along with increased systemic vascular resistance in patients with Cushing’s disease in comparison with those in control group, which confirms an unfavorable hemodynamic profile in Cushing’s disease patients (pronounced vasoconstriction and impaired left ventricular hemodynamic function). The observed subclinical hemodynamic abnormalities support an additive effect of long-term hypercortisolemia on cardiovascular function in patients with Cushing’s disease. One of the most common adverse complications of long-term tissue exposure to excess glucocorticoids is arterial hypertension, which develops in over 70% of patients with Cushing’s disease and is an independent risk factor for mortality in this group of patients (15, 16, 18, 19). Interestingly, the hypothalamic–pituitary–adrenal axis, which is responsible for the circadian rhythm of endogenous cortisol secretion, also contributes to the regulation of the circadian blood pressure rhythm, and its dysregulation is one of the main factors associated with primary arterial hypertension (20–22). Clinical measurements of arterial hypertension in patients with Cushing’s disease may be difficult in cases of hypercortisolemia. Current guidelines stress the need for personalized antihypertensive treatment (18, 23, 24) since arterial hypertension is an independent risk factor for mortality in patients with Cushing’s disease (18, 19, 25). There is a linear relationship between arterial hypertension and both adverse cardiovascular events and mortality (25). Therefore, early detection of cardiovascular complications (including arterial hypertension, even before it becomes clinically manifest) may be of clinical significance and may help reduce cardiovascular mortality in patients with Cushing’s disease. Out of the 54 patients with Cushing’s disease in our study 64.8% were diagnosed with arterial hypertension. The pathophysiology of arterial hypertension in Cushing’s disease patients is multifactorial and not fully understood. Arterial hypertension in these patients seems to be an effect of both hypercortisolism and increased activation of the renin–angiotensin–aldosterone system (23, 24, 26–29). These complex processes result in secondary endothelial dysfunction (4, 30–32) and increased carotid artery intima–media thickness, which is associated with atherosclerotic plaque developing earlier than in healthy individuals (15, 30, 33). Therefore, the increased arterial stiffness and abnormal vasoconstriction may play a key role in the pathophysiology of arterial hypertension in this patient population (34).

Our observations regarding this issue, based on the data obtained in our study with the use of a noninvasive method, i.e. impedance cardiography, are consistent with those reported by other authors. The results of our study showed that patients with Cushing’s disease have considerably higher values of afterload indicators, which suggests that vasoconstriction abnormalities play a fundamental role in arterial hypertension pathophysiology in these patients, despite their relatively well-controlled blood pressure. This indicates that a hemodynamic assessment with impedance cardiography has an added value, which has been also suggested by the results of other studies. For instance, normal systolic blood pressure values in a group of heart failure patients were not equivalent to an optimal hemodynamic status. This is because systemic vascular resistance index may be increased and contribute to progressive myocardial remodeling even with low systolic blood pressure values of (100–119 mmHg) (11). This is important for antihypertensive therapy selection in this group of patients and indicates a fact of enormous practical significance, namely, that routine blood pressure measurements may be insufficient to rule out hemodynamic dysfunction (11). Thus, the following types of vasodilators would seem to be indicated as first-line therapy in this group of patients: angiotensin converting enzyme inhibitors, angiotensin receptor blockers, and calcium channel blockers; this is consistent with the results of other studies (23, 26, 35).

Moreover, the results of our study indicate that patients with Cushing’s disease have lower thoracic fluid content than controls. Thoracic fluid content accurately reflects the amount of intra- and extracellular fluid and is a sensitive indicator of fluid retention and hypervolemia (8). Our study observations regarding this parameter are not entirely consistent with those reported by other authors. Development of arterial hypertension in patients with Cushing’s disease may be associated with an increased mineralocorticoid activity of glucocorticoids, enhanced reabsorption of sodium in the renal tubules, and—consequently—increased intravascular volume (23, 24). Some reports emphasize that this does not seem to be the main pathophysiological mechanism responsible for arterial hypertension in patients with Cushing’s disease (23). The low thoracic fluid content values observed in our study also support this conclusion. The combination of a low cardiac index (an indirect measure of intravascular volume) and normal left ventricular ejection fraction in patients with Cushing’s disease seems to be an argument against the use of diuretics as a first-line antihypertensive therapy.

Our study also showed that patients with Cushing’s disease had lower values of cardiac function parameters (stroke index, velocity index, cardiac index, acceleration index) in comparison with controls. This may be explained by rapid remodeling and hypercortisolism-induced fibrosis of the myocardium (17, 36). Patients with Cushing’s disease often exhibit evidence of structural remodeling of the myocardium, associated with concentric left ventricular hypertrophy (17). The presence of both arterial hypertension and hypercortisolemia in patients with Cushing’s disease considerably worsens the structural and functional status of their myocardium (37, 38). They develop myocardial fibrosis, which is directly related to the effects of cortisol and not merely a result of myocardial hypertrophy due to pressure overload (36). These structural changes may impair left ventricular hemodynamic function, which first manifests as diastolic dysfunction and, subsequently, as systolic dysfunction and development of symptomatic heart failure (17, 39, 40). One prospective study demonstrated that a successful curative treatment normalized cortisol levels and ultimately led to resolution of myocardial remodeling (41). A study in patients with heart failure showed a significant correlation between changes in the cardiac index value determined *via* impedance cardiography and the left ventricular ejection fraction measured echocardiographically (11). Moreover, a hemodynamic assessment was reported to be of clinical significance in patients with heart failure with preserved left ventricular systolic function (42). There was also a significant correlation between impaired left ventricular systolic function and low values of parameters characterizing blood flow, such as velocity index and acceleration index (43). Other studies suggest that, in patients with arterial hypertension, impedance cardiography may be a useful method for assessing left ventricular dysfunction, whose important predictors are cardiac index (p=0.005) and systemic vascular resistance index (p=0.048) (9). Earlier studies demonstrated the usefulness of impedance cardiography in assessing left ventricular dysfunction and increased arterial stiffness in middle-aged and elderly patients. Left ventricular diastolic dysfunction was shown to be associated with a lower stroke index, velocity index, acceleration index, and Heather index and a higher systemic vascular resistance index (10). Also, echocardiographic evidence of impaired left ventricular global longitudinal strain was reported to be associated with a hemodynamic profile similar to that found in patients with Cushing’s disease (low cardiac index; high systemic vascular resistance index) (44). These correlations were also confirmed in patients with Cushing’s disease, in whom an impaired left ventricular global longitudinal strain was associated with left ventricular diastolic dysfunction and were detectable at early stages of pituitary disease (45).

### Clinical Implications

This study showed that impedance cardiography can be more sensitive than routine blood pressure measurements and may be a valuable early method of detecting subclinical left ventricular dysfunction in Cushing’s disease. Impedance cardiography results indicating early cardiovascular hemodynamic abnormalities despite normal blood pressure values may be an additional argument for initiating early therapeutic intervention, even in cases where the diagnosis of arterial hypertension is uncertain. Moreover, detecting an impaired cardiac function may prompt a more intensive treatment in patients on antihypertensive medications.

### Study Limitations

The main limitation of our study was the relatively small sample size. This is a result of low Cushing’s disease incidence but also of the study’s prospective design. At the time of their diagnosis, many patients with Cushing’s disease exhibit signs of significant cardiovascular dysfunction. However, this was an exclusion criterion in our study, which further reduced the study population. Moreover, the patients with Cushing’s disease included in this study had no clinically overt cardiovascular dysfunction, and any individuals with severe comorbidities were excluded at the time of recruitment. When interpreting study results, we should consider the potential effects of arterial hypertension (despite its good control) and of antihypertensive treatment. The potential effect of the sex of patients with Cushing’s disease on their hemodynamic dysfunction requires further studies.

## Conclusions

1. Hormonal disorders associated with Cushing’s disease led to cardiovascular dysfunction manifesting as impaired cardiac function, low indices of myocardial contractility, low thoracic fluid content, and increased systemic vascular resistance.
2. Assessing Cushing’s disease patients with the use of impedance cardiography may be useful in detecting early cardiovascular complications and help make decisions as to early introduction of medical treatment.

## Data Availability Statement

The datasets presented in this study can be found in online repositories. The names of the repository/repositories and accession number(s) can be found in the article/supplementary material.

## Ethics Statement

The studies involving human participants were reviewed and approved by Bioethics Committee of Military Institute of Medicine in Warsaw. The patients/participants provided their written informed consent to participate in this study.

## Author Contributions

Study concept and design, data acquisition and interpretation, and correction of the manuscript: AJ, GG, PK, BU-Ż, PW, GZ, AK, RW, and MB. Data analysis and editing of the manuscript: AJ, PK, GG, BU-Ż, and PW. All authors contributed to the article and approved the submitted version.

## Funding

This study was financed by government-allotted funds dispensed by the Military Medical Institute in Warsaw (WIM/MNiSW grant No. 453/WIM).

## Conflict of Interest

The authors declare that the research was conducted in the absence of any commercial or financial relationships that could be construed as a potential conflict of interest.

## Publisher’s Note

All claims expressed in this article are solely those of the authors and do not necessarily represent those of their affiliated organizations, or those of the publisher, the editors and the reviewers. Any product that may be evaluated in this article, or claim that may be made by its manufacturer, is not guaranteed or endorsed by the publisher.
